# Eating Jet Lag: A Marker of the Variability in Meal Timing and Its Association with Body Mass Index

**DOI:** 10.3390/nu11122980

**Published:** 2019-12-06

**Authors:** María Fernanda Zerón-Rugerio, Álvaro Hernáez, Armida Patricia Porras-Loaiza, Trinitat Cambras, Maria Izquierdo-Pulido

**Affiliations:** 1 Department of Nutrition, Food Science and Gastronomy, School of Pharmacy and Food Science, University of Barcelona, 08028 Barcelona, Spain; fernanda.zeron@ub.edu; 2 INSA-UB, Nutrition and Food Safety Research Institute, University of Barcelona, 08921 Santa Coloma de Gramenet, Spain; 3 Cardiovascular Risk, Nutrition and Aging Research Unit, August Pi i Sunyer Biomedical Research Institute (IDIBAPS), Barcelona, 08036 Spain; alvaro.hernaez1@gmail.com; 4 CIBER Physiopathology of Obesity and Nutrition (CIBEROBN), Instituto de Salud Carlos III, 28029 Madrid, Spain; 5 Department of Health Sciences, Universidad de las Americas Puebla, 72810 Puebla, Mexico; patricia.porras@udlap.mx; 6 Department of Biochemistry and Physiology, School of Pharmacy and Food Science, University of Barcelona, 08028 Barcelona, Spain; cambras@ub.edu

**Keywords:** meal timing, eating jet lag, body mass index, obesity, young adults

## Abstract

The timing of food intake has been associated with obesity and adverse metabolic outcomes, independently of the amount or content of food intake and activity level. However, the impact of the variability in the timing of food intake between weekends and weekdays on BMI (body mass index) remains unexplored. To address that, we propose to study a marker of the variability of meal timing on weekends versus weekdays (denominated as ‘eating jet lag’) that could be associated with increments in BMI. This cross-sectional study included 1106 subjects (aged 18–25 years). Linear regression models were used to examine the associations of eating jet lag with BMI and circadian related variables (including chronotype, eating duration, sleep duration, and social jet lag). Subsequently, a hierarchical multivariate regression analysis was conducted to determine whether the association of eating jet lag with BMI was independent of potentially confounding variables (e.g., chronotype and social jet lag). Moreover, restricted cubic splines were calculated to study the shape of the association between eating jet lag and BMI. Our results revealed a positive association between eating jet lag and BMI (*p* = 0.008), which was independent of the chronotype and social jet lag. Further analysis revealed the threshold of eating jet lag was of 3.5 h or more, from which the BMI could significantly increase. These results provided evidence of the suitability of the eating jet lag, as a marker of the variability in meal timing between weekends and weekdays, for the study of the influence of meal timing on obesity. In a long run, the reduction of the variability between meal timing on weekends versus weekdays could be included as part of food timing guidelines for the prevention of obesity among general population.

## 1. Introduction

Eating is a complex behavior with a variety of personal, social, cultural, and environmental factors playing a role in *what* and *when* we eat [1,2,3]. The latter constitutes an important aspect of nutrition that has been causally related to obesity and adverse metabolic outcomes. Importantly, it has been found that this association is independent of dietary intake (energy and nutrients) and the level of physical activity [4,5]. In particular, the delay of the timing of food intake has been associated with decreased basal energy expenditure and diet-induced thermogenesis, as well as impaired glucose tolerance [1,4,6,7]. Moreover, a statement from the American Heart Association has pointed out that irregular meal patterns, such as day-to-day inconsistencies in the timing and frequency of meals, could also be unfavorable with respect to weight status and cardiometabolic [8]. Several biological mechanisms have been described to explain these associations, including the role of the circadian system [1].

The circadian system is comprised by a master clock and a network of peripheral clocks, all of which are organized in a hierarchical manner [9,10]. While the master clock is located in the suprachiasmatic nucleus of the hypothalamus and regulates main body functions (e.g., core body temperature, blood pressure or sleep), peripheral clocks are located in almost all tissues of the body (including liver, pancreas, muscle, and adipose tissues) and regulate many metabolic processes (e.g., metabolism and glucose homeostasis) [11]. Similar to an orchestra conductor, the master clock uses the inputs from the light-dark cycle to determine the time of day, and impose temporal order to the body’s peripheral clocks. In this way, the master clock coordinates behavioral rhythms such as sleep–wake and feeding–fasting cycles, and thus, it organizes a sequence of physiological processes to optimize metabolism, mainly through peripheral clocks [12].

Noteworthy, peripheral clocks can be synchronized by the timing of food intake and, thus, it could alter internal synchrony between the central and the peripheral clocks. The latter is known as internal misalignment, and is related to adverse metabolic outcomes [8,9,13]. Eating during the usual sleeping period (known as mistimed feeding) is an example of internal misalignment [14,15]. In this case, the concurrence between the postprandial period and melatonin, would impair insulin secretion, and thus, glucose tolerance [16]. Additionally, irregular eating timing could dampen diurnal circadian rhythms, especially those involved in the anticipatory response to feeding [12], leading to the impairment of glucose homeostasis, changes in energy expenditure or gastrointestinal alterations [12,15,17].

The anticipatory response to feeding is in charge of regulating nutrient homeostasis during the postprandial period [12]. Consequently, when feeding occurs at the expected (or usual) time, the circadian clock ensures that the appropriate pathways that help to assimilate the nutrients begin to rise in anticipation of feeding. In this way, the organism can handle nutrient utilization, and therefore, homeostasis is maintained [12]. Thus, food consumed within a consistent interval appears to sustain optimal nutrient utilization and promote health [12]. However, when feeding occurs at an unexpected (or unusual) time, nutrient sensing pathways act on the peripheral clocks, so that in the subsequent days food is anticipated at the new feeding time [12,18]. Thereby, feeding can independently activate nutrient-sensing pathways, compromising the way how food is processed during the postprandial period.

In free-living populations, that is, subjects living outside the laboratory, shiftwork is known to be the most extreme situation for internal misalignment. However, a small but chronic version of shiftwork is social jet lag, in which the discrepancy in the sleep/wake schedules between weekdays and weekends induce a mild kind of misalignment [19]. Social jet lag has been considered as a potential risk factor for metabolic health and obesity [19,20,21,22]. In addition, social jet lag has been associated with unhealthy eating habits, as it was recently shown by our research group [23]. Although, the variability in meal timing has been scarcely examined, a recent study has demonstrated that regular breakfast consumption pattern is an important factor in maintaining a healthy weight [24]. In line with this, Panda et al. [25] showed that eating patterns largely differed between weekdays and weekends in young adults. Of note, both authors suggested that irregular breakfast consumption would trigger internal misalignment. Furthermore, in Guinter’s [24] study, internal misalignment was actually used as a potential mechanism to explain the association between breakfast irregularity and obesity [24,25]. Taking into account the aforementioned, we hereby propose a novel marker of the variability in meal timing, which, due to its resemblance to social jet lag [26], we have denominated ‘eating jet lag’. Our aim was to study the association of eating jet lag with the body mass index (BMI) in a sample of young healthy adults. We hypothesized that a high variability in meal timing on weekends versus weekdays (eating jet lag) would be associated with higher BMI.

## 2. Materials and Methods

### 2.1. Study Design, Settings, Participants, and Protocol

Subjects aged 18–25 years were recruited among undergraduate and postgraduate students in Spain at the University of Barcelona, and in Mexico at the Universidad de las Americas Puebla. Individuals were invited to participate in this cross-sectional study during the school year, between 2017 and 2019. Recruitment consisted of an informative talk, explaining details to the participants about the research, and encouraging them to enroll the study. Exclusion criteria consisted of the inability to provide information required for the development of the study or being previously diagnosed with chronic diseases such as type 2 diabetes, hypertension, and/or cardiovascular disease. Based on these criteria, a total of 1121 individuals were eligible and provided written informed consent before joining this study. We further excluded subjects with missing information, resulting in a final analytical cohort of 1106 subjects (72.1% Spanish). All study procedures were conducted according to the Declaration of Helsinki and were approved by Ethics Committee the University of Barcelona and by the Ethics and Research Committee of the Department of Health Sciences of the Universidad de las Americas Puebla.

### 2.2. Eating Period

Habitual timing of breakfast, lunch and dinner was estimated in weekends and weekdays by questionnaire. In this case, participants were asked: ‘During weekdays/weekends: At what time do you usually eat breakfast’, ‘During weekdays/weekends: At what time do you usually eat lunch?’ and ‘During weekdays/weekends: At what time do you usually eat dinner?’

From these data we calculated the following variables:

(i) Average eating duration. Eating duration was defined as the length between the first and the last caloric event (in hours) [27]. To calculate average eating duration, first, we estimated the eating duration for weekdays and weekends as follows: Eating duration (h) = Timing of the last meal − Timing of the first meal. Subsequently, we estimated the average eating duration as a weighted mean as follows: Average eating duration (h) = [(5 × eating duration on weekdays) + (2 × eating duration on weekends)]/7.

(ii) Eating midpoint, defined as the middle time point between the first and the last meal. This parameter was calculated for weekdays and weekends based on the methodology proposed to estimate the midpoint of sleep [28]. Thus, the eating midpoint was calculated as follows: Eating midpoint (local time) = ([Timing of the last meal − Timing of the first meal]/2) + Timing of the first meal.

### 2.3. Eating Jet Lag

This parameter was defined as the variability of the timing of the eating period, and was estimated following the methodology proposed to calculate social jet lag [26]. Accordingly, eating jet lag was calculated in hours as follows: Eating midpoint on weekends − Eating midpoint on weekdays.

Additionally, the variability in the timing of breakfast, lunch or dinner was calculated as follows: Breakfast or lunch or dinner jet lag (h) = Time on weekends − Time on weekdays. All analyses were conducted using the absolute value of the estimated eating, breakfast, lunch, or dinner jet lag [26].

Integer values of eating, breakfast, lunch, or dinner jet lag were used to evaluate the frequency of the delay or advance of each meal timing on weekends. Thereby, “advance” in the timing of a meal was considered if values were lower than −1, “delay” in the timing of a meal was considered if values higher than +1, and the “maintenance” in the timing of the meal was considered if values ranged from −1 to +1 [25].

### 2.4. Anthropometric Parameters

Self-reported height and weight were queried in a questionnaire as ‘What is your current weight? (kg)’ and ‘What is your current height? (cm)’. BMI was calculated as weight (kg) divided by squared height (m).

### 2.5. Sleep and Circadian Parameters

The chronotype was estimated using the phase (local time) of the middle between bed and wakeup timing (midpoint of sleep) on free days (MSF), based on the instructions accompanying the Munich Chronotype Questionnaire [28].

Social jet lag (h), defined as the discrepancy between the internal and external timing, was measured by subtracting each participant’s midpoint of sleep on weekdays, from the midpoint of sleep on weekends [26]. All analyses were conducted using the absolute value of social jet lag [22,26].

Habitual sleep duration (h) was estimated by questionnaire including the questions ‘During weekdays/weekends: At what time do you usually go to bed?’, ‘During weekdays/weekends: At what time do you usually wake up?’. A total weekly sleep duration was calculated as follows: [5 × weekday sleep (h) + 2 × weekend sleep (h)]/7 [29].

### 2.6. Lifestyle Variables

Diet quality was evaluated through the Mediterranean Diet Quality Index (KIDMED) [30] for Spanish young subjects. The KIDMED scores range from −4 to 12 and has demonstrated reliable psychometric properties using Chronbach’s alfa (α = 0.765). Diet quality for Mexican subjects was evaluated through the Quality Index Food Consumption Pattern (QIFCP) [31]. In this case, scores range from 0 to 93. Additionally, the QIFCP demonstrated reliable psychometric properties using Chronbach’s alfa (α = 0.762). In both cases, the higher the score, the better the diet quality. Thus, scores were transformed into z-scores and then combined in a common variable of “diet quality”. Noteworthy, diet quality was evaluated with the appropriate tool for each country because of the differences in typical products and crops, as well as, the diversity in cultural, geographical, and ecological environments between the two countries.

Finally, physical activity was measured with the short version of the International Physical Activity Questionnaire (IPAQ) [32]. Physical activity was evaluated in minutes per week of Metabolic Equivalent of Task (MET), for both populations. In this case, the higher the score, the more intense the level of physical activity. This questionnaire demonstrated reliable psychometric properties using Chronbach’s alfa (α = 0.713). Additionally, it has been validated in a similar population and has demonstrated a good correlation with accelerometer data [32].

### 2.7. Statistical Analyses

Normality was confirmed in all variables by histograms and Q-Q plots. Variables were described by means and standard deviations for continuous variables and proportions for categorical data. Paired t-tests were used to compare the mean of the timing of breakfast, lunch, dinner and eating duration on weekends versus weekdays. Associations between eating, breakfast, lunch and dinner jet lag with circadian related variables and BMI were studied using Pearson correlations. Any significant relationship between variables was further investigated with linear regression analyses and adjusted by covariates. In addition, a hierarchical multivariate regression analysis was conducted to determine whether eating jet lag was associated with BMI, independent of potentially confounding variables. Analyses were performed with the SPSS statistical computer software, version 24.0 (IBM SPSS Statistics, Armonk, NY, USA). Restricted cubic splines were used to analyze the shape of the association between eating jet lag and BMI. We set the reference cut-point at the minimum observed value of the eating jet lag (0 h). Splines were fitted and plotted using the “glm’ package in R Software (R Foundation for Statistical Computing, Vienna, Austria) [33]. All models were adjusted for age, gender, nationality, physical activity, sleep duration, and diet quality. A statistical test was considered significant when *p* < 0.05.

## 3. Results

Briefly, 1106 individuals (72% Spanish; 78% females; 21.0 ± 2.5 years) were included in this cross-sectional study (Table S1). Overall, 16.4% of the individuals had overweight or obesity, while 58.0% of the individuals showed an average diet quality and 50.7% reported vigorous physical activity. In addition, mean chronotype (shown by the MSF) was at 05:17 ± 01:13 h and mean social jet lag was of 1.7 ± 1.0 h.

### 3.1. Differences in the Timing of Food Intake between Weekends and Weekdays among Young Adults

Regarding the timing of food intake, significant differences were found in the timing of breakfast, lunch, and dinner and in the eating midpoint between weekdays and weekends (*p* < 0.001) (Table 1). Interestingly, the highest discrepancy in meal timing between weekends and weekdays was at breakfast (2.0 h ± 1.2 h). Concerning eating jet lag, our results revealed that almost two thirds of the population studied (64%) showed more than 1 h of eating jet lag, out of which 22.5% had more than 2 h of eating jet lag.

**Figure 1:**
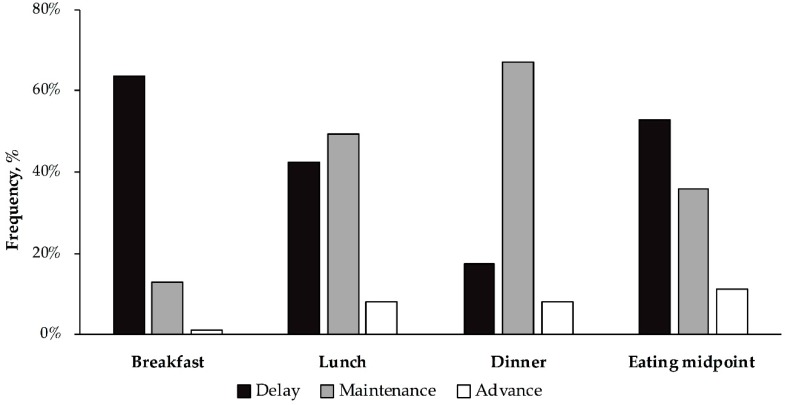
Frequencies of the delay, maintenance, or advance on meal timing during among the population studied. Values represent the percentage of individuals delaying, maintaining or advancing each meal timing. The black bars indicate the delay in the timing of a meal, gray bars indicate the maintenance on the timing of the meal, and white bars indicate the advance in the timing of a meal.

Moreover, we analyzed the frequency of the: delay, maintenance or advance, of the timing of each meal (breakfast, lunch, dinner) and the eating midpoint on weekends versus weekdays (Figure 1). In which case, we observed that the participants usually delayed the timing of breakfast and the eating midpoint on weekends, whereas the timing of lunch was either maintained or delayed and dinner timing was mostly maintained during weekends.

Interestingly, the variability of meal timing on weekends versus weekdays was associated with circadian variables (Table 2). In particular, the chronotype and social jet lag were significantly associated with breakfast, lunch, dinner, and eating jet lag. Accordingly, a higher tendency towards the evening and/or greater social jet lag would be associated with a greater variability in meal timing on weekends versus weekdays.

### 3.2. Increments in the Eating Jet Lag Are Associated with Higher BMI Values among the Population Studied

Results from the linear regression models revealed that neither breakfast, lunch or dinner jet lag were significantly associated with BMI. Noteworthy, eating jet lag was positively and significantly associated with BMI (*p* = 0.001). Thus, individuals who showed higher eating jet lag also showed greater BMI. In addition, we conducted a hierarchical multivariate regression analysis to study whether the association between eating jet lag and BMI was independent of potentially confounding variables (Table 3). As shown in Model 2, after controlling for nationality, age, gender, physical activity, diet quality, and sleep duration, the association between eating jet lag and BMI remained significant (β = 0.280, *p* = 0.006). Moreover, Model 3 showed that the association between BMI and eating jet lag was independent of the chronotype and social jet lag (β = 0.283, *p* = 0.008). Overall, according to adjusted R^2^ coefficients from the models, Model 2 fitted the data better than the other two models. Among other findings, our results showed that the nationality, age, and gender of the individuals were also significant predictors of BMI.

**Figure 2:**
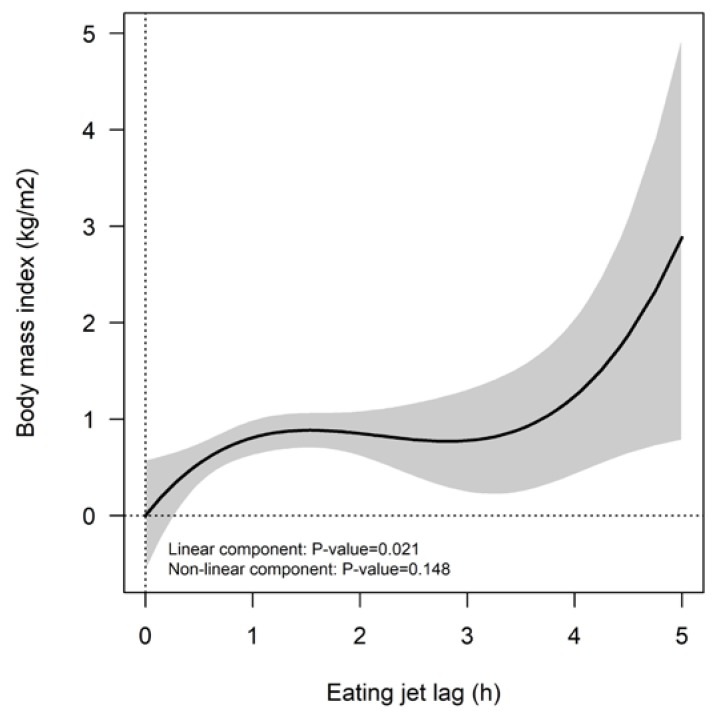
Restricted cubic spline model of the association between eating jet lag and the BMI. BMI, Body mass index. Cubic spline models adjusted by age, gender, nationality, physical activity, diet quality, and sleep duration. The gray band indicates the confidence levels for the regression line.

Finally, restricted cubic splines were used to study the shape of the association between eating jet lag and BMI (Figure 2). Noteworthy, we observed a threshold of 3.5 h or more of eating jet lag, from which the BMI could significantly increase. Specifically, our data showed that having more than 3.5 h of eating jet lag was associated with higher BMI values (1.34 kg/m^2^ [95% CI: 0.026; 2.40]; *p* = 0.015).

## 4. Discussion

As far as we are aware, this is the first study to investigate the variability in meal timing between weekends and weekdays, in a large sample of free-living young adults from two different countries and cultural backgrounds. Our results indicated that the shift in breakfast timing was the most pronounced (on average 2 h), whereas lunch and dinner jet lags were less evident (on average 1 h and 0.5 h, respectively). Two other studies have previously reported on the variability of breakfast, lunch, and dinner timing during the weekend, both in small samples of young adults (*n* = ~100) [5,25]. In consonance with our findings, the authors pointed out that delaying the timing of the meals (especially breakfast) was common among young adults. Interestingly, Gill and Panda [25] suggested that the changes in breakfast timing during the weekend could induce some kind of metabolic desynchrony. Nonetheless, the researchers failed to associate the variability of breakfast timing on weekends with BMI. Moreover, due to the absence of a clear meal pattern, Gill and Panda suggested that the eating duration could be a better description of an individual’s eating pattern [25].

Taking into account the aforementioned, we defined the middle time point of the eating period (eating midpoint), which allowed us to define a novel marker of variability of meal timing between weekends and weekdays, denominated “eating jet lag”. In the population studied, the eating jet lag was, on average, of 1.3 h and it was related with the chronotype and social jet lag. Importantly, we observed that higher eating jet lag was significantly associated with higher BMI. This association was independent of the nationality, age, gender, physical activity level, diet quality, sleep duration, and eating duration, as well as chronotype and social jet lag.

As a plausible mechanism, similar to the circadian desynchrony arising from social jet lag and its impact on weight [26], we postulate that eating jet lag could be linked to internal misalignment. It is important to highlight that a specific timing system (defined as food clock by Challet [15]) tracks predictable changes on energy status (via circulating nutrients and hormonal inputs) and drives rhythmic behavior in anticipation to food availability, adjusting the phase of the peripheral clocks, but not the master clock [1,9,15]. Noteworthy, experimental studies on mice have shown that when feeding is limited to the resting phase, the food clock drives changes in some variables, including plasma glucocorticoids and body temperature. Subsequently, these components become uncoupled from the regular nocturnal pattern, leading to obesity and dysmetabolism [15,17].

Although experimental evidence in humans is limited, a recent study showed that postponing the timing of the first meal (5.5 h) resulted in the delay of both plasma glucose and *PER 2* rhythms in the adipose tissue, without altering the rhythm of the master clock [34]. As a conclusion, the authors highlighted the role of meal timing as a time-giver for peripheral clocks [34]. Moreover, evidence from forced desynchrony protocols has shown that the misalignment between feeding/fasting and the master clock reduced energy expenditure, as well as leptin and peptide YY levels. To add, this misalignment led individuals to a prediabetic state, characterized by elevated post-prandial glucose and insulin [17,35].

At a population level, the strongest evidence of circadian and metabolic misalignment is night shift-workers. Evidence from two meta-analyses suggests that shift-workers are more likely to have obesity [36], regardless of the amount of daily energy intake [37]. The authors implied that circadian misalignment, as well as the timing and distribution of energy intake, might be underlying the overweight and obesity development in this population [36,37]. Noteworthy, our data revealed that the variability in the timing of food intake on weekends versus weekdays was also associated with overweight. In this regard, we found a threshold of eating jet lag of 3.5 h or more, from which increments in the BMI could be observed. In particular, our data showed that BMI was 1.34 kg/m^2^ higher in subjects who reported more than 3.5 h of eating jet lag. Although more evidence needs to be warranted, it is important to highlight that this variability in the timing of food intake on weekends versus weekdays could occur chronically during an individual’s life. Thus, future studies should evaluate the impact of the chronic variability in meal timing on weight evolution as well.

Among other findings, our data showed that neither breakfast, lunch, or dinner jet lag were associated to BMI. In which case, we hypothesize that the regularity in the timing of the first and the last meal has a potential role in the temporal regulation of metabolism, and thus, in weight. In this regard, lessons from time-restricted-feeding interventions in humans have taught that food consumed within a consistent 8–12 h period appeared to sustain optimal nutrient utilization and promote health [12]. Interestingly, a 16-week intervention in young adults showed that reducing the duration of the eating period from 14 h to 10–12 h was related to weight loss (~1.15 kg/m^2^) and regularity in breakfast timing [25]. Noteworthy, time-restricted-feeding helps to temporally separate incompatible biochemical processes (e.g., biosynthesis and degradation of a given molecule), thereby preventing futile cycles [38]. Furthermore, it is plausible that when energy intake is aligned with energy expenditure, and clear feeding/fasting cycles are synchronized with metabolic changes, robust circadian rhythms would be maintained and, consequently, health would be promoted [10].

Recently Challet [15] pointed out that the hormonal cues induced by food intake after a meal (“post-feeding timers”) could have a potential role in the synchronization of the circadian clock. For example, meal-induced secretion of insulin could reset peripheral clocks by acting at transcriptional and post-transcriptional levels in the liver. Furthermore, incretins such as glucagon-like peptide-1 (GLP-1), which are secreted during the post-prandial period, can change the phase of peripheral clocks as well. Although more evidence needs to be warranted to unravel the effects of these post-feeding time cues, these endocrine signals seem to be important time-givers related to food intake [15].

On the other hand, our results showed that the variability in meal timing was associated with the chronotype and social jet lag. Interestingly, we observed that breakfast, lunch, and dinner and eating jet lag were significantly associated with chronotype. Therefore, evening-type subjects (‘owls’) present a higher variability in meal timing during weekends versus workdays. Noteworthy, it has been pointed out that individual chronotype leads the preference of the timing of different activities, including sleeping and eating [39,40]. In particular, eveningness is characterized by a preference towards later sleep/wake timing, thus, we hypothesize that this delay in the timing of food intake on weekends would be in line with their circadian preference [19,21,41]. Although the chronotype is known to be a modifier of the relationship between meal timing and obesity [40], our results showed that chronotype did not influenced the association between the variability of meal timing on weekends versus weekdays and a higher BMI. Nonetheless, we cannot ignore the interactions between environmental, cultural, and social factors that could be decisive factors in the choice of meal timing [1,40,41].

As mentioned above, social jet lag was also associated with the variability in meal timing on weekends versus weekdays. Usually, individuals who suffer from social jet lag sleep longer on weekends to recover from the sleep debt accumulated through the week [19,26]. Thus, delayed wakeup timing would be consequently associated with later timing of food intake on weekends. Interestingly, Roenneberg et al. [19] previously demonstrated that the chronic disruption between the biological clock and the social clock was associated with obesity. As a conclusion, the authors suggested that improving the correspondence between the biological and the social clock will contribute to the management of obesity. Noteworthy, when eating jet lag and social jet lag were included in the multivariate regression model as potential predictors of BMI, eating jet lag was significantly associated with BMI, while social jet lag was not. Thus, it is plausible that the variability in meal timing (masked by the social jet lag) could be triggering the misalignment between peripheral and central clocks. The latter could indicate that the regularity in eating schedules should be considered in health recommendations.

Furthermore, it is important to note that a well-known drawback in the study of meal timing and its role in obesity is the lack of a consistent approach to define meal timing [3,39,42]. Several authors have mentioned that it is difficult to evaluate the impact of the timing of food intake across populations, mainly because conventional meal categories (e.g., breakfast, lunch, and dinner) differ within cultures and people tend to skip meals through the week [3,39,42]. Thus, in agreement with other authors [25], in the current study we propose that the duration of the of the eating period could be used as an adequate description of an individual’s eating pattern. Moreover, we have gone further and suggested the ‘eating midpoint’ as a proxy to evaluate the variability in meal timing through the week. Thus, eating midpoint could be used to evaluate the impact of the variability in the timing of food intake on health, and even in different populations.

This study has certain limitations to consider when interpreting our findings: first, its cross-sectional nature prevented us to find causation. Second, we acknowledge our results are based upon a cohort of young healthy adults, which may not be representative of the entire population in terms of sleep and meal timing. Hence, future studies should evaluate the impact of eating jet lag in other populations, including different age groups, socioeconomic status, as well as subjects with different metabolic conditions. Third, we acknowledge the use of self-reported measurements (BMI, self-reported bed and wakeup time, and meal timing) as a limitation of the study. Nonetheless, our study has certain strengths including the large sample size, which provided sufficient power to evaluate the variability on meal timing on weekends versus weekdays and its influence on BMI. In addition, the use of two different populations reinforced the effect of eating jet lag on BMI. However, more evidence needs to be warranted regarding the effects of eating jet lag on different biomarkers, including those related to metabolic syndrome or hunger related hormones. Additionally, it would be interesting to evaluate the potential association of the eating jet lag with dietary intake (energy and nutrients), as well as the frequency of food intake and other habits like late night snacking or TV viewing while eating. Furthermore, subsequent studies should examine the potential association between eating jet lag and other determinants of the timing of food intake (e.g., social or cultural activities) on weekends. Finally, we encourage future experimental studies to objectively evaluate the effect of eating jet lag on metabolic health, as well as to unravel the mechanisms underlying the association between eating jet lag and higher BMI.

## 5. Conclusions

In conclusion, the results from this study showed that eating jet lag can be a suitable marker of the variability of meal timing on weekends versus weekdays, which would potentially be related to internal misalignment. Additionally, we have demonstrated that greater eating jet lag associates with higher BMI in young adults. Hence, the study of eating jet lag could provide the framework for future intervention studies in the field of obesity and related metabolic diseases. To focus on the regularity in meal timing on weekends and weekdays as part of weight-loss trials seems essential in order to elaborate future chrononutritional recommendations for the general population and for health promotion.

## Figures and Tables

**Table 1:** Differences in meal timing between weekends and weekdays in the population studied.

Meal Timing Variables	Mean (SD)
Breakfast	
Weekdays, hh:mm	08:21 (01:15)
Weekends, hh:mm	10:24 (01:08)
*p*-value ^a^	**<0.001**
Breakfast jet lag, h	2.0 (1.2)
Lunch	
Weekdays, hh:mm	14:15 (01:09)
Weekends, hh:mm	14:53 (00:58)
*p*-value *^a^*	**<0.001**
Lunch jet lag, h	0.9 (0.8)
Dinner	
Weekdays, hh:mm	21:17 (00:47)
Weekends, hh:mm	21:32 (00:56)
*p*-value ^a^	**<0.001**
Dinner jet lag, h	0.5 (0.7)
Eating midpoint	
Weekdays, h	15:09 (01:28)
Weekends, h	16:01 (01:18)
*p*-value ^a^	**<0.001**
Eating jet lag, h	1.3 (0.9)

SD, Standard deviation. Data are expressed as mean (SD). ^a^ Paired *t*-tests were used to compare the mean of the timing of breakfast, lunch, and dinner and eating duration on weekends versus weekdays. Significant *p*-values < 0.05 are shown in bold.

**Table 2:** Associations between breakfast, lunch, and dinner and eating jet lag with circadian related variables.

	Chronotype (MSF)			Social Jet Lag		
	β	95% CI	*p*-Value ^a^	β	95% CI	*p*-Value ^a^
Breakfast jet lag	0.320	0.250, 0.0390	**<0.00001**	0.720	0.640, 0.800	**<0.00001**
Lunch jet lag	0.100	0.061, 0.141	**<0.00001**	0.049	0.002, 0.096	**0.042**
Dinner jet lag	0.073	0.061, 0.140	**<0.00001**	0.072	0.038, 0.110	**<0.001**
Eating jet lag	0.110	0.063, 0.160	**<0.00001**	0.270	0.210, 0.330	**<0.00001**

MSF, Midpoint of sleep on free days; CI, confidence interval. Data was analyzed using linear regression models to test associations between breakfast, lunch, and dinner and eating jet lag with continuous outcome measures of chronotype and social jet lag. The table shows the unstandardized coefficient (β), CI and *p*-value associated with each predictor variable. ^a^ Analyses were conducted with age, gender, nationality, diet quality, sleep duration, and physical activity as covariates. Significant *p*-values are shown in bold.

**Table 3:** Hierarchical multivariate regression analysis of predictors of the body mass index.

Variables	Model 1	Model 2	Model 3
	β (95% CI)	β (95% CI)	β (95% CI)
Eating jet lag, h	0.336 (0.132, 0.540) **	0.280 (0.080, 0.479) **	0.283 (0.073, 0.494) **
Nationality (1, Spanish; 2, Mexicans)		1.671 (1.232, 2.111) **	1.621 (1.129, 2.113) ***
age, years		0.239 (0.162, 0.317) ***	0.237 (0.159, 0.316) ***
Gender (1, male; 2, females)		−1.076 (−1.550, −0.603) ***	−1.067 (−1.546, −0.588) ***
Physical activity, METs		0.000 (0.000, 0.000)	0.000 (0.000, 0.000)
Diet quality, z-score		−0.012 (−0.206, 0.182)	0.013 (−0.192, 0.218)
Sleep duration, h		−0.081 (−0.281, 0.120)	−0.094 (−0.300, 0.111)
Average eating duration, h			−0.033 (−0.120, 0.054)
Chronotype, MSF			0.031 (−0.207, 0.268)
Social jet lag, h			−0.034 (−0.320, 0.253)
R^2^	0.009 **	0.089 ***	0.087 ***
R^2^ change	0.009 **	0.085 ***	0.001
F change	10.479	17.111	0.242

CI, Confidence Interval; h, hours; METs, Metabolic Equivalents of Task; MSF, Midpoint of sleep on free-days. Multivariate regression analyses were used to test the association of eating jet lag with BMI. The table shows the unstandardized coefficient (β), CI and *p*-value associated with each predictor variable. Significant *p*-values ** <0.01, *** <0.001.

## Supplementary Materials

The following are available online at https://www.mdpi.com/2072-6643/11/12/2980/s1, Table S1: General characteristics of the studied population.
