# Erratum: Eating Jet Lag: A Marker of the Variability in Meal Timing and its Association with Body Mass Index

**DOI:** 10.3390/nu12030816

**Published:** 2020-03-19

**Authors:** María Fernanda Zerón-Rugerio, Álvaro Hernáez, Armida Patricia Porras-Loaiza, Trinitat Cambras, Maria Izquierdo-Pulido

**Affiliations:** 1 Department of Nutrition, Food Science and Gastronomy, School of Pharmacy and Food Science, University of Barcelona, 08028 Barcelona, Spain; fernanda.zeron@ub.edu; 2 INSA-UB, Nutrition and Food Safety Research Institute, University of Barcelona, 08921 Santa Coloma de Gramenet, Spain; 3 Cardiovascular Risk, Nutrition and Aging Research Unit, August Pi i Sunyer Biomedical Research Institute (IDIBAPS), 08036 Barcelona, Spain; alvaro.hernaez1@gmail.com; 4 CIBER Physiopathology of Obesity and Nutrition (CIBEROBN), Instituto de Salud Carlos III, 28029 Madrid, Spain; 5 Department of Health Sciences, Universidad de las Americas Puebla, 72810 Puebla, Mexico; patricia.porras@udlap.mx; 6 Department of Biochemistry and Physiology, School of Pharmacy and Food Science, University of Barcelona, 08028 Barcelona, Spain; cambras@ub.edu

The Nutrients Editorial Office would like to update the error in the original published version [1].

The affiliation of the fifth author, “Maria Izquierdo-Pulido ^2,4,^*^,†^”, has been changed to “Maria Izquierdo-Pulido ^1,2,4,^*^,†^, who also belongs to the Department of Nutrition, Food Science and Gastronomy, School of Pharmacy and Food Science, University of Barcelona, 08028 Barcelona, Spain.” 

We would like to apologize for any inconvenience caused to the authors and readers by this mistake. We will update the article, and the original version will remain available on the article webpage.
