# Supplementary material for: Eating Jet Lag: A Marker of the Variability in Meal Timing and Its Association with Body Mass Index
Source: Nutrients. 2019 Dec 6;11(12):2980. doi: 10.3390/nu11122980 (PMC6950551; doi:10.3390/nu11122980)
Supplement: Supplementary file 1 [file nutrients-11-02980-s001.pdf]

Table S1. General characteristics of the studied population.

**Variable**	Mean (SD)
Sample size, n	1 106
% Spanish	72.1
% Females	78.4
Age, years	21.0 (2.5)
**BMI**, kg/m^2	22.2 (3.4)
Underweight, %	8.0
Normal-weight, %	75.5
Overweight, %	12.9
Obese, %	3.5
**Diet quality**	
Low, %	7.6
Average, %	58.0
High, %	34.4
**Physical Activity**	
Low, %	22.2
Moderate, %	27.0
Vigorous, %	50.7
**Circadian related variables**	
Sleep duration, h	8.1 (1.0)
Chronotype (MSF), hh:mm	05:17 (01:13)
Social jet lag, h	1.7 (1.0)
Eating duration, h	11.3 (2.5)

Data are expressed as mean (SD) or proportions. Abbreviations: BMI, body mass index; hh:mm, hours: minutes; MSF, midpoint of sleep on free-days.
